# Localised and sustained intradermal delivery of methotrexate using nanocrystal-loaded microneedle arrays: Potential for enhanced treatment of psoriasis

**DOI:** 10.1016/j.ejps.2020.105469

**Published:** 2020-09-01

**Authors:** Ismaiel A. Tekko, Andi Dian Permana, Lalitkumar Vora, Taher Hatahet, Helen O. McCarthy, Ryan F. Donnelly

**Affiliations:** a School of Pharmacy, Queen's University Belfast, Medical Biology Centre, 97 Lisburn Road, Belfast, BT9 7BL, Northern Ireland, United Kingdom; b Department of Pharmaceutics and Pharmaceutical Technology, Faculty of Pharmacy, Aleppo University*,* Aleppo, Syria; c Department of Pharmaceutics, Faculty of Pharmacy, Hasanuddin University*,* Makassar*,* Indonesia

**Keywords:** Methotrexate, Psoriasis, Intradermal drug delivery, Nanocrystals, Dissolving microneedles, Sustained release

## Abstract

Methotrexate (MTX), typically used as its sodium salt (MTX Na), is a first-line treatments for moderate to severe psoriasis, showing good efficacy. However, its systemic administration is associated with many side effects. Intradermal delivery into psoriatic tissue could offer an alternative approach. However, successful intradermal administration of MTX Na is currently precluded by its physicochemical properties. Moreover, due to its hydrophilic nature, MTX Na is swiftly cleared from the target tissue, necessitating frequent dosing which may affect patient compliance. To address these limitations, we investigated the combination of nanocrystal (NC) and dissolving microneedle (MN) technologies as an alternative approach for localised and sustained intradermal delivery of MTX Na. Poorly water-soluble MTX nanocrystals (MTX NC) were produced by a bottom-up technique with a mean particle size of 678 ± 15 nm. Sustained *in vitro* drug release was observed over 72 h. The MTX NC were then incorporated into the shafts of dissolving MN arrays with a drug loading of 2.48 mg/array. The MTX NC-loaded MN arrays exhibited satisfactory mechanical strength and insertion capabilities in the skin-simulant Parafilm M® and their shafts dissolved entirely in less than 20 min after insertion into excised neonatal porcine skin. Importantly, *in vivo* studies in Sprague Dawley rats revealed that the MN arrays were able to deposit approximately 25.1% of the loaded MTX NC in the skin, which acted, in turn, as a drug depot and released the MTX in a sustained manner over 72 h, while minimising MTX systemic exposure. Indeed, 24 h from MN application, 312.70 ± 161.95 µg/g of MTX was retained in the skin at the application site. This was approximately 322-fold higher than the amount of MTX (0.942 ± 0.59 µg/g) retained in the skin after oral administration of MTX Na. Interestingly, even after 72 h after MN application, around 12.5% of the MTX NC deposited in the skin by the MN was retained. In contrast, the maximal blood concentration of MTX achieved following MN application, was only 40% of that measured after oral administration of MTX Na. Accordingly, MTX NC-loaded dissolving MN arrays could be a promising approach for effective localised and sustained intradermal delivery of MTX as a potential enhanced treatment for psoriasis.

## Highlights

• We developed methotrexate nanocrystals for its localising and sustaining its release in at the application site in the skin.
• We incorporated the nanocrystals into dissolving microneedles for intradermal delivery.
• We successfully localised and sustained methotrexate release in the skin compared to conventional oral route.
• We successfully reduced the systemic methotrexate exposure using our novel approach.

## Graphical abstract

**Figure:**
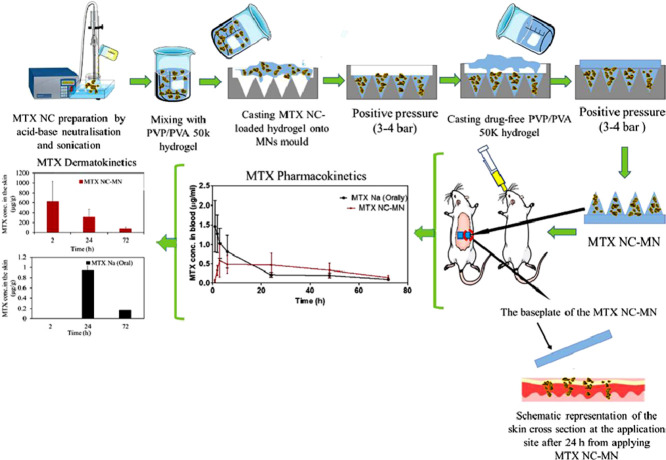

## 1 Introduction

Psoriasis is a chronic inflammatory autoimmune skin disease that affects 1–3% of the population, with approximately 125 million people worldwide being affected (ODaly, 2012; Parnami et al., 2014). Around 50–140 new cases per 100,000 people are reported every year (Raho et al., 2012; Pinto et al., 2014). This disease is challenging to treat and it is characterised by infiltration of the leucocyte into the skin, leading to the development of scaling erythematous plaques, along with skin shedding, swelling, itching, and painful inflammation, which affect the patient`s quality of life and limits his social interactions (Parnami et al., 2014; Dubey et al., 2007; Mukhtar et al., 2004). The exact cause of psoriasis is still unclear. Despite being studied for many decades with some notable achievements, no firm conclusions as to the aetiology and pathogenesis of this disease have been reached (Zhao et al., 2020).

Currently several treatment strategies are in use, including photochemotherapy with UVA (PUVA), retinoids, cyclosporin A and methotrexate (MTX) (Raho et al., 2012). MTX is a folate antagonist that is known for its anti-inflammatory and immune-modulatory effects (Cabello Zurita et al., 2017; Gyulai et al., 2015). This drug was approved by the USA FDA to treat psoriasis in the early 1970s (Avasatthi et al., 2016). Despite the significant advances in psoriasis therapy today, MTX remains in clinical use and is listed as one of the first options in multiple treatment guidelines for moderate to severe psoriasis (Czarnecka-Operacz and Sadowska-Przytocka, 2014; Du et al., 2019; West et al., 2016). The most common chemical form of the drug that is used in therapy is its sodium salt (MTX Na). MTX Na is administered systemically, either orally or parenterally at a dose of 7.5–20 mg once weekly (Gyulai et al., 2015).

Even though MTX Na shows a remarkable antipsoriatic effect and is more cost-effective than other therapies (Cabello Zurita et al., 2017; Kim et al., 2017), its current administration routes and drug delivery systems are not so satisfactory. Systemic administration of MTX Na leads to prolonged high levels of systemic drug exposure, which are associated with many side effects, including gastrointestinal disturbances, such as nausea, vomiting and diarrhoea, hepatotoxicity, suppression of bone marrow function, dyspnoea, leukopenia, anaemia, thrombocytopenia and menstrual alteration (Branco et al., 2016; Bianchi et al., 2016; Jacobse et al., 2019; Braun et al., 2008; Braun and Rau, 2009; Koçak et al., 2013; Boukhettala et al., 2009; Wang et al., 2018). Moreover, orally administered MTX Na suffers from first-hepatic metabolism and nonlinear pharmacokinetics, which affect its bioavailability, consequently its clinical efficacy (Hoekstra et al., 2006). Parenteral administration using disposable needles and syringes, as an alternative administration route, is inconvenient and stressful, which lead to poor patient compliance (Jacobse et al., 2019; Bechard et al., 2014). Additionally, MTX Na is highly water-soluble and has a relatively short half-life; thus it is eliminated rapidly from the body, which may affect its efficacy (Bello et al., 2017) and necessitate more frequent doing (Du et al., 2019). In clinical practice, MTX Na is known as a slow-acting drug as it may take several weeks before patients can notice the desired therapeutic effects (Vena et al., 2018). To address these limitations, an alternative administration route and drug delivery system are highly desirable.

Intradermal drug delivery, which is defined as bypassing the outermost layer of the skin, the *stratum corneum*, by a suitable device and depositing the drug in the underlying viable skin layers, the epidermis and dermis, is an attractive alternative drug delivery approach employed to overcome certain drug administration challenges (Zhao et al., 2020; Larrañeta et al., 2016). Indeed, delivering MTX directly to the psoriatic tissue has several advantages include (i) sparing a high proportion of the systemically administered dose (Du et al., 2019), (ii) reducing the unnecessary systemic MTX exposure, thus avoiding/reducing its systemic toxicity and (iii) evading the non-linear pharmacokinetics and the variable bioavailability associated with oral delivery, thus improving drug efficacy (Prasad and Koul, 2012; Chen et al., 2019; Gupta et al., 2012). Despite these advantages, intradermal delivery of MTX Na into psoriatic tissue faces significant challenges in that MTX Na is a hydrophilic drug (log P = -1.85) and is mostly in the dissociated form at physiological pH. Accordingly, its passive permeation across the outermost layer of the skin, the *stratum corneum* is very limited (Prasad and Koul, 2012; Tekko et al., 2006; Nguyen and Banga, 2018; Weinstein et al., 1989). For example, in one study, Weinstein and co-workers have reported that the passive penetration of MTX Na from an aqueous drug solution (2% MTX) was around 5 µg/cm^2^ after 48 h (Weinstein et al., 1989). To overcome the poor passive permeation of MTX Na, several strategies have been investigated, such as employing niosomes, liposomes, nanogels, electroporation, sonophoresis and iontophoresis (Prasad and Koul, 2012). Despite increased drug permeation, it is still challenging to administer a sufficient dose of MTX Na for optimal disease management using any of these methods (Du et al., 2019; Prasad and Koul, 2012).

Microneedle (MN) arrays are minimally invasive devices composed of small projections (less than 1 mm in length) which can by-pass the *stratum corneum* without pain to form small aqueous pores through which drugs deposited in or penetrate through to the dermal microcirculation (Gomaa et al., 2012; Ramöller et al., 2019). MN can be fabricated from various materials, such as silicon, metal, ceramic and dissolving polymers (Ita, 2015). To exploit this novel delivery system in the administration of MTX Na intradermally (*i.e.* into the skin), various MN array types and designs have been investigated. MN made of maltose, silicon, and poly(D, L-lactic-co-glycolic acid) (PLGA) have greatly enhanced the skin permeability to MTX Na *in vitro* (Abla et al., 2013; Vemulapalli et al., 2008; Nguyen and Banga, 2018). Nevertheless, the therapeutic effects have yet to be tested *in vivo*. It is worth noting that in these studies a two-step “poke and patch” approach is used, by which MN merely are employed to generate microchannels in the skin before the application of a topical MTX Na formulation. This approach has shown to be burdensome and error-prone (Ita, 2015). Additionally, the micropores generated by MN are quickly closed, resulting in reduced drug permeation. Disturbingly, silicon MN remain solid after removal from a patient's skin which may impose hazards of inappropriate re-use.

Rapidly dissolving MN arrays have attracted considerable interest over the past two decades, since they are composed of water-soluble polymers that incorporate the drug within the MN themselves (Ita, 2015). Upon insertion into skin, they completely dissolve or degrade in the skin without leaving any sharps or biohazardous materials. Additionally, dissolving MN require one step-application (Ita, 2015). Recently, MTX Na-loaded dissolving MN prepared from a biodegradable polymer, the hyaluronic acid, has shown to be a promising drug delivery system to treat psoriasis. Not only because they can dissolve quickly and deliver their payload directly to the psoriatic site, but also they spare a high proportion of the dose (Du et al., 2019). However, the loading dose was quite low and frequent daily dosing was necessary to maintain the therapeutic drug effect (Du et al., 2019), which may affect patient compliance and adherence to the therapy. This may be ascribed to the fact that the drug used was MTX Na, which is freely water-soluble (dos Santos et al., 2017). Thus, upon application, it is swiftly cleared from the application site, which may affect efficacy. Additionally, MTX Na has a short half-life, approximately 4.5–10 h (Palakurthi et al., 2011; Bello et al., 2017).

A novel dissolving MN combined with high drug loading and sustained or kinetically-controlled drug release capability would be highly desirable. Such a drug delivery system would have several advantages including (i) maintaining drug levels at the application site within the therapeutic window over several days, consequently improving drug efficacy and reducing the necessity for frequent drug dosing, thus improving patient compliance and (ii) reducing the unnecessary MTX Na systemic exposure, thus avoiding/reducing toxicity/side effects.

Several strategies for obtaining MN with sustained drug release capability could possibly be employed, including formulation of the highly water soluble drug into biodegradable polymeric micro- or nanoparticles (Battisti et al., 2019; Ventre et al., 2018) or using one of the insoluble forms of the drug (either its free acid for acid drugs or free base for basic drugs), which they can be then incorporated into the dissolving MN shafts to form a sustained release drug delivery system. Unfortunately, using biodegradable polymeric micro- or nanoparticles often necessitates high amounts of polymeric excipients to encapsulate the drug. Clearly, this results in a lower drug loading in the MN arrays (Battisti et al., 2019; Vora et al., 2018). Alternatively, a commercially available MTX free acid (MTX), which is the poorly water-soluble form of the drug could be used. However, this poorly water-soluble drug form exists as micron-sized crystals and incorporating such drug crystals into a hydrophilic polymeric hydrogel (which forms the MN matrix) has been reported to be a challenging task, due to the difficulty of micron-sized hydrophobic drug-polymeric hydrogel mixing. This could lead to production of inhomogeneous MN with variable drug loadings (Vora et al., 2018; Mc Crudden et al., 2018, Abdelghany et al., 2019). Water-miscible organic solvents can be used to facilitate drug mixing with the MN polymeric matrix hydrogel. However, using such organic solvents may affect the mechanical properties of the MN, thus reducing skin insertion capability (Vora et al., 2018).

NC (interchangeably also called a nanosuspension) are unique sub-micron colloidal dispersions of pure drug particles stabilised using small amounts of surfactants and/or polymers. NC typically have particle sizes of 1–1000 nm (Müller and Peters, 1998). NC represent a relatively new type of colloidal delivery system employed initially to enhance dissolution and bioavailability of poorly water-soluble drugs (Müller and Peters, 1998). Recently, our Group has used this technology in combination with dissolving MN to form a novel drug delivery system that successfully delivered several poorly water soluble drugs into the skin including curcumin, vitamin D and rilpivirine (Vora et al., 2018; Mc Crudden et al., 2018; Abdelghany et al., 2019). Using this approach, NC helped in incorporating the hydrophobic drugs homogeneously into the polymeric MN matrix. Importantly, upon intradermal delivery by the MN, the NC acted as a depot, sustaining drug release for days and even weeks *in vivo* (Vora et al., 2018; Mc Crudden et al., 2018; Abdelghany et al., 2019). Additionally, since NC formulation requires only small amounts of stabiliser, they yield high drug loadings in the MN arrays (Zhai et al., 2014). This study investigated, for the first time, the combination of NC and dissolving MN technologies as an alternative approach for localised and sustained intradermal delivery of MTX, with a view to enhanced treatment for psoriasis and minimise/avoid the side effects associated with its systemic administration.

## 2 Materials and methods

### 2.1 Materials

Methotrexate disodium (MTX Na) (purity ≥ 99.35%) was purchased from Haihang Industry Co. LTD (Shandong, China). Poly(vinylalcohol) (MW= 9–10 kDa, 80% hydrolysed) (PVA 10 K) and poly(vinylalcohol) (MW=31–50 kDa, 87–89% hydrolysed) (PVA 50 K) were purchased from Sigma-Aldrich (Dorset, UK). Poly(vinylpyrrolidone) (MW=58 kDa) (PVP) was obtained from Ashland (Kidderminster, UK). Ultrapure water was obtained from a water purification system (Elga PURELAB DV 25, Veolia Water Systems, Dublin, Ireland). All other reagents used were of analytical grade.

### 2.2 Preparation of methotrexate nanocrystals (MTX NC)

**Fig. 1:**
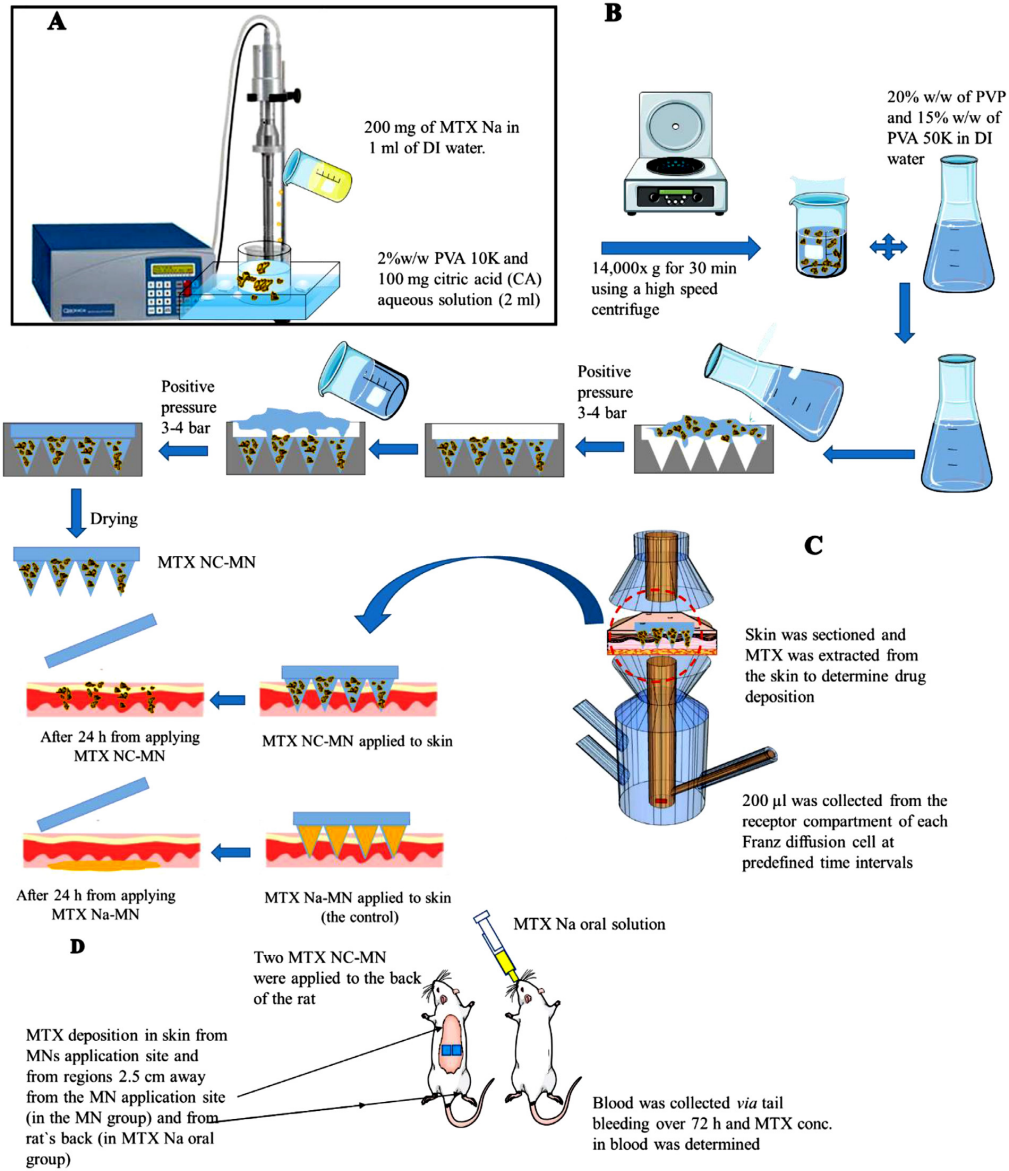
Schematic diagram of (A) Preparing MTX NC by the acid-base neutralisation precipitation method under sonication; (B) Preparation procedures of dissolving bilayer MTX NC-loaded MN by two-step method; (C) The experimental set up for the ex vivo studies to evaluate MTX dermatokinetic and release kinetics after inserting either MTX NC-MN or MTX Na-MN (the control) into an excised full-thickness neonatal porcine skin mounted between the two compartments of Franz diffusion cells; (D) The experimental set-up for *in vivo* studies which were performed to evaluate MTX pharmacokinetics and dermatokinetics in Sprague Dawley rats following MTX administration using MTX NC-MN and MTX oral solution.

MTX NC were prepared by employing a bottom-up technique, namely the acid-base neutralisation precipitation method with sonication previously described (dos Santos et al., 2017; Mou et al., 2011), with minor modifications as depicted in Fig. 1A. Briefly, PVA 10 K and citric acid (CA) were used as a stabiliser and pH modifier, respectively. Initially, 200 mg of MTX Na (equivalent to 182.2 mg/ml of MTX free acid) was dissolved in 1 ml deionised (DI) water resulting in an MTX Na aqueous solution with a pH of 7.88. Separately, 2 ml of an aqueous solution containing 2% w/w PVA 10 K (the stabiliser) and 100 mg of CA was prepared. To precipitate MTX particles, the drug solution (1 ml) was slowly titrated by syringe over 3 min into the stabiliser solution (2 ml) while sonicating using a probe sonicator (QS4 system, NanoLab, Waltham, MA, USA), which was then continued for another 2 min at an amplitude of 80% (of 125 W, 20 kHz) with 10-sec pulse on and 5-sec pulse off. The temperature of the stabiliser solution was maintained at 5° ± 3  °C during sonication using an ice bath. To remove the excess water and produce a concentrated MTX NC dispersion, the resultant dispersion was spun at 14,000 x g for 30 min using a high-speed centrifuge (Sigma™ 1–14 micro-centrifuge, SciQuip Ltd., Shropshire, UK). Subsequently, 2 ml of the drug-free supernatant solution was removed and discarded. The concentrated MTX NC dispersion was then collected and stored at 2- 8  °C until further use.

### 2.3 Fabrication of dissolving bilayer MTX NC- MN and needle-free patches

To minimise drug wastage and to load the drug in the MN shafts only, bilayer MTX NC-loaded MN were prepared by two-step casting method using a positive pressure chamber. Initially, 40% w/w PVP and 30% w/w PVA 50 K hydrogels were made separately and then mixed at a ratio of 50:50, w/w to produce a polymeric blend (containing 20% w/w PVP and 15% w/w PVA 50 K) which was used to form the matrix for both layers of the MN arrays. The PVP/PVA 50 K polymeric blend was homogeneously mixed with a stainless-steel spatula for 10 min and then centrifuged at 3500 rpm for 15 min to remove any air bubbles. The MTX NC-loaded hydrogel formulation (used to prepare the first MN array layer) was prepared by mixing the PVP/PVA 50 K blend with the concentrated MTX NC dispersion prepared in Section 2.2. To investigate the possibility of maximising drug loading in the MN arrays, the polymeric blend and the concentrated MTX were mixed at various mixing ratios as per Table 1. The same PVP/PVA 50 K polymeric blend (but contains no drug) was used to cast the second MN layer.

**Table 1:** Composition of the MTX NC-loaded hydrogel used to cast the first MN layer.

Formulation	Concentrated MTX NC: PVP/PVA 50 K hydrogel, mixing ratio (w/w)
F1	1:9
F2	2.5:7.5
F3	5:5
F4	7.5:2.5

Bilayer dissolving MN arrays were prepared as illustrated in Fig. 1B. Briefly, an aliquot of approximately 50 mg of the MTX NC-loaded hydrogel of each of the above-mentioned formulations was cast into the MN mould prepared from poly(dimethyl siloxane) (PDMS) and consisting of 16×16 square-based pyramidal needle holes (each needle hole has a height of 850 µm and 300 µm side length at the baseplate). The moulds were then placed in the positive pressure chamber, and a pressure of 3–4 bar was applied for 3 min to fill the needles cavities in the moulds before being taken out and removing the formulation excess. The moulds were placed again in the pressure chamber for 15 min under the same pressure. Subsequently, the moulds were removed from the pressure chamber and allowed to dry at ambient room temperature for 2 h. To cast the second MN layer and baseplate, an aliquot of approximately 200 mg of the drug-free PVP/PVA 50 K polymeric blend was cast onto the top of the first MN layer of each mould. The moulds were then placed again in the pressure chamber pressure at 3–4 bar for 10 min and then removed. Finally, due to MTX photosensitivity, The MN were allowed to dry by placing the moulds in the dark at 37 ± 1  °C in a lab incubator (Genlab Ltd, Cheshire, UK) for 48 h. After drying, the bilayer MTX NC-MN were then gently peeled from the moulds. The MTX NC-MN were placed in 12-well Nunc plate, covered with an aluminium sheet and stored in a desiccator under vacuum until further use.

As a control, dissolving bilayer MN arrays (MTX Na-MN) loaded with the freely-water soluble MTX Na with a drug content equivalent to finalised MTX NC-MN (with highest possible drug loading) were also prepared using the same procedures as above. Needle-free patches were also prepared to determine the density of the MTX NC-MN shafts in the dry state to estimate the drug content per MN array. To prepare the needle-free patches, in triplicate, an aliquot of 1000 mg of each MTX NC-loaded hydrogel formulation (F1, F2, F3 and F4) was cast into 2×2 cm in-house customised moulds, spread homogenously and then were again allowed to dry in the lab incubator for 48 h. Subsequently, the needle-free patches were removed, weighed, and stored after covering with an aluminium sheet in a desiccator under vacuum until further use.

### 2.4 Characterisation of the MTX NC

#### 2.4.1 Particle size, PDI and zeta potential measurement

The particle size, polydispersity index (PDI) and zeta potential of MTX NC formulation were determined using a NanoBrook Omni particle size and zeta potential analyser (Brookhaven, New York, USA). Before measurement, the concentrated MTX NC samples were diluted (1: 99, v/v) with DI water. All analyses were carried out in triplicate at ambient room temperature (25 ± 2  °C).

#### 2.4.2 X-ray diffraction measurements

The X-ray measurements were carried out using a benchtop X-ray diffractometer (Miniflex™, Rigaku, Neu-Isenburg, Germany) equipped with Ni-filtered, Cu Kβ radiation, at a voltage of 30 kV and a current of 15 mA. The concentrated MTX NC was first dried in a lab incubator (Genlab Ltd, Cheshire, UK) at 37 ± 1  °C for 48 h and then pulverised using stainless-steel beads and a high-speed sample homogeniser (TissueLyser LT, Qiagen Corp, Manchester, UK) for 60 s. The plain PVA 10 K, MTX Na and the pulverised MTX NC were respectively packed into the rotating sample holder and analysed individually. The obtained data were typically collected by scanning at a range of 3–45° in a continuous mode with a scanning rate of 2°/min.

#### 2.4.3 Scanning electron microscopy

Scanning electron microscopy (SEM) was used to examine the morphology of the particles. Samples from the concentrated MTX NC which was prepared for X-ray diffraction measurements were sprinkled onto a sticky carbon tape, sputter-coated at 2.5 kV, 18 mA with gold for 45 s (POLARON E5150, Gold Sputter Coater, Quorum Technologies, East Sussex, UK) and viewed under SEM using Quanta FEG 250 benchtop scanning electron microscope (SEM) (FEI, Hillsboro, OR, USA) at an acceleration voltage of 10–20 kV under high chamber pressure (8 × 10^−5^ mbar).

#### 2.4.4 Drug content determination

Drug content was determined by diluting an aliquot of 100 µl of the concentrated MTX NC with 9.9 ml of 0.1% w/w NaOH aqueous solution, then vortexed for 5 min until complete dissolution before being filtered through 0.45 µm PTFE non-sterile syringe filter. The filtrate was analysed for MTX content using the validated HPLC-UV method described below in the pharmaceutical analysis section.

#### 2.4.5 In vitro drug release evaluation

MTX release from its MTX NC and MTX Na solution (as a control) was evaluated *in vitro* using a dialysis method (Permana et al., 2019). To mimic the conditions of the interstitial fluid in the skin, phosphate-buffered saline 10 mM (PBS, pH 7.4) was used as a release medium. In triplicate, 1 ml of either the MTX Na solution or the MTX NC dispersion (each containing drug equivalent to 5 mg MTX free acid) were loaded into the Spectra-Por®, 12,000–14,000 MWCO dialysis bag (Spectrum Medical Industries, Los Angeles, CA, USA). The bags were each then suspended in 100 ml of the PBS release medium at 37 ± 1 C in an orbital shaker at 100 rpm over 72 h. At predetermined time intervals, aliquots of 1 ml were collected and replaced with a new pre-equilibrated release media solution. To calculate the amount of drug released, MTX was quantified in the collected samples using the validated HPLC-UV method described below in the pharmaceutical analysis section.

To determine the release model that best described the drug release kinetics, the *in vitro* release data was substituted into different mathematical release kinetic models, namely zero-order, first-order, Higuchi and Korsmeyer- Peppas, as outlined in previous studies (Permana et al., 2019).

$$\text{Zero}-\text{order}:Q_{t}=Q_{0}+K_{0}t$$

$$\text{First}-\text{order}:lnQ_{t}=\text{ln}0_{o}+\;K_{1}t$$

$$\text{Higuchi}:Q_{t}=K_{H}\sqrt{t}$$

$$\text{Korsmeyer}-\text{Peppas}:Q_{t}=K_{\text{kp}}n$$

Where Q_t_ (%) is the percentage of drug released at the time t, Q_0_ is the starting value of Q_t_, t is the time, n is the diffusion release exponent, K_0_, K_1_, K_h_ and K_kp_ are the release constants corresponding to relevant kinetic models. Finally, the release kinetic model parameters were calculated using DD solver (Zhang et al., 2010).

### 2.5 Characterisation of the bilayer dissolving MTX NC- MN

#### 2.5.1 Drug content determination

To calculate the amount of MTX localised in the needles of the MTX NC-MN array, the following equation was used (Permana et al., 2019):

(1) $$\mathrm{Drug}\;\mathrm{content}\;\mathrm{in}\;\mathrm{the}\;\mathrm{MN}\;\mathrm{array}=n.\frac{\left(h.a^{2}\right).\rho .\left[\mathrm{drug}\right]}{3}$$

Where *n* is the number of needles per MN array (*n* = 256), *a* is the side length base of the tips (*a* = 0.3 mm), *h* is the height of the needle (0.800 mm), *ρ* is the MN density in the dry state, and *[drug]* is the concentration of MTX in the needle-free patch (mg drug/mg patch). Both *ρ* and *[drug]* were determined from the needle-free patches which were prepared in Section 2.3. as follows:

The needle-free patches prepared from each formulation were weighed in, and their dimensions measured by a digital micro-calliper and used to calculate *ρ. [drug]* was determined by dissolving a pre-determined weight of the needle-free patch in 10 ml of 0.1% NaOH, vortexed for 10 min and then filtered through 0.45 µm PTFE non-sterile syringe filters. The filtrates were then analysed for MTX content using the validated HPLC-UV method described below in the pharmaceutical analysis section.

#### 2.5.2 Assessment the effects of MN polymers and fabrication process on MTX NC properties

To evaluate the effects of MN polymers and fabrication process on MTX NC properties, MTX NC-MN were dissolved in 10 ml of DI water. After appropriate dilution, particle size, PDI and zeta potential of MTX NC were measured as described in Section 2.4.1.

#### 2.5.3 Mechanical and insertion properties of MTX NC-MN assessment

A TA.XT2 Texture Analyser (Stable Micro Systems, Haslemere, UK) in compression mode was used to determine mechanical and insertion properties of bilayer MTX NC- MN, as previously described by our Group (Ramöller et al., 2019; Vora et al., 2018; Permana et al., 2019; Larrañeta et al., 2014). Briefly, the MN was attached to a cylindrical probe (cross-sectional area 1.5 cm²), and the Texture Analyser arm moved vertically downward at a speed of 0.5 mm/s and the MN compressed against a flat aluminium block. A force of 32 N was applied for 30 s before the probe was moved upward again. The length of individual needles in the MN arrays was measured before and after compression using Leica EZ4W stereo microscope with an integrated camera (Leica Microsystems, Milton Keynes, UK). The percentage reduction of MN heights was calculated and reported.

To evaluate the insertion properties of MTX NC- MN, the same set up as before was used (speed 0.5 mm/s, force 32 N). However, MN were moved downward to be compressed against a previously validated artificial skin-simulant membrane consisting of a stack of 8 layers of Parafilm M® (Bemis Inc., Soignies, Belgium) (Larrañeta et al., 2014). The Parafilm M® layers were then separated, and the holes created in each layer were counted using a Leica EZ4W stereo microscope.

The MTX NC-MN which had the maximum possible drug loading and maintained satisfactory mechanical and insertion properties were selected as the finalised MTX NC-MN formulation and then subjected to further testing.

To increase our confidence in the ability of our MN to be inserted into the skin, the insertion of the finalised MTX NC-MN into excised full-thickness neonatal porcine skin, which is a good model for human skin (Larrañeta et al., 2014; Summerfield et al., 2015), was also evaluated using the same experimental set up as before. Skin samples were obtained from stillborn piglets. They were stored immediately after birth at

-20  °C and thawed for two days before being skinned. Full-thickness skin was cut into pieces and stored at -20  °C until further use. Skin samples were equilibrated in PBS 10 mM (pH 7.4) for 30 min and shaved before use The needle insertion into the skin was monitored using an EX1301 optical coherence tomography (OCT) microscope (Michelson Diagnostics Ltd., Kent, UK), as described previously (Larrañeta et al., 2014) and the insertion depth measured using the imaging software ImageJ® (National Institute of Health, Bethesda, MD, USA). A light microscope was also used to visualise the needles residuals in the skin after 20 min from MN application.

#### 2.5.4 Ex vivo dissolution kinetic study

Neonatal porcine skin samples were prepared and equilibrated, as described previously (Mc Crudden et al., 2018; Abdelghany et al., 2019; Permana et al., 2019; Meyer, 1996) and in Section 2.5.3., and then placed on tissue paper soaked in PBS (pH 7.4) to maintain skin hydration and pinned to a Styrofoam™ platform wrapped in aluminium foil. The finalised MTX NC-MN were then inserted manually into the skin using a 5 ml syringe plunger. After applying pressure for 30 s, a 13.0 g cylindrical stainless-steel block was placed on top of each MN array, keeping them in place. MTX NC-MN arrays were removed from the skin at pre-determined time intervals (0, 5, 10, 15 and 20 min). Then, the height of the MN was measured using the Leica EZ4W stereo microscope and the percentage needle height reduction was determined.

#### 2.5.5 Ex vivo MTX dermatokinetic and release studies

These studies were performed to evaluate the ability of the MTX NC to retain the drug at the application site in the skin and sustain its release, following their delivery by the MN (Permana et al., 2019). These studies were achieved by studying MTX deposition and distribution in the skin and its release from the skin following inserting of the finalised MTX NC-MN arrays into an excised full-thickness neonatal porcine skin (*ex vivo*) which was mounted into a Franz diffusion cells, as previously described (Permana et al., 2019). Illustration of the experimental set up is shown in Fig. 1C.

PBS 10 mM (pH 7.4) solution was used in the receiver compartment and was thermostatically held at 37 ± 1  °C with stirring at 600 rpm to mimic the skin temperature and the pH of the interstitial fluid in the skin. The finalised MTX NC- MN were then inserted into the skin using manual force for 30 s, and a cylindrical stainless-steel block of 13.0 g again was placed on top to hold the MN in place. To prevent water evaporation, the donor compartment and sampling arm were wrapped using Parafilm M®. An aliquot of 200 µl was collected from the receptor compartment of each Franz diffusion cell at pre-defined time intervals of 0.5, 0.75, 1, 2, 3, 4, 5, 6, 7, 8 and 24 h and then replenished with an equal volume of a fresh pre-equilibrated PBS solution. The concentrations of MTX were quantified using the validated HPLC-UV method described below in the pharmaceutical analysis section.

With respect to the dermatokinetics study, this was performed as previously described (Permana et al., 2019). Briefly, an experimental set up similar to that employed in the drug release study was again used. Again, the finalised MTX NC- MN were then inserted into the skin using manual force for 30 s and the cylindrical stainless-steel block of 13.0 g was again placed on top to hold the MN in place. At predefined time intervals (2, 4, 8 and 24 h), the applied MN were removed, and the skin was washed three times with PBS (pH 7.4) to remove any excess MN formulation. Skin samples at the application site were then taken using a biopsy punch (5.0 mm diameter) (Stiefel, Middlesex, UK). Following this, the samples were quickly frozen in liquid nitrogen, then mounted on Finalised Cutting Temperature Medium® (Sakura, Thatcham, UK) with the epidermis side facing up. These samples were finally cryostat-cut into 200 µm-thick sections using a Leica CM1900 Cryostat (Leica Microsystems Ltd, Milton Keynes, UK). An aliquot (500 µl) of methanol: water (1:1, v/v) was added to the individual tissue sections in 1.5 ml Eppendorf tubes, which were then vortexed for 20 min to dissolve the drug. Following this, samples were centrifuged at 14,000×g for 15 min, and the supernatant was finally collected and then analysed using the validated HPLC-UV method described below in the pharmaceutical analytical section.

For comparison, MN arrays loaded with the freely water-soluble MTX Na (MTX Na-MN) (with drug content equal to the drug content of the finalised MTX NC-MN arrays) was also prepared and similarly tested and used as a control.

### 2.6 *In vivo* pharmacokinetics and dermatokinetics studies

This study aimed to evaluate the efficiency of MTX NC-MN in delivering the drug into the skin and the ability of MTX NC to retain the drug in the skin and sustain its release *in vivo*. For comparison, orally administered MTX Na aqueous solution was used as a control. The experimental set up is depicted in Fig. 1D.

#### 2.6.1 Experimental design and drug administration procedures

This *in vivo* study was conducted according to the policy of the Federation of European Laboratory Animal Science Associations and the European Convention for the protection of vertebrate animals used for experimental and other scientific purposes, with the implementation of the principles of the 3Rs (replacement, reduction, and refinement). Approval was obtained from the Committee of the Biological Research Unit, Queen's University Belfast to carry out this study under Project Licence PPL 2794 and Personal Licences PIL 1747 and 1890.

The study was performed using healthy female Sprague-Dawley rats (n = 23) aged between 14- 16 weeks and their average weight was 251.0 ± 35.4 g. Rats were allowed to acclimatise to laboratory conditions for at least seven days before the experiment and then split into two cohorts. Cohort 1 (used as a control) (n = 10), each rat received 0.5 ml of MTX Na aqueous solution (1.25 mg/ml, calculated as MTX free acid) by gastric gavage, which corresponds to a dose of 2.5 ± 0.15 mg/kg. Cohort 2 (n = 13), each rat received two of the finalised MTX NC-MN (each MN contains 2.48 ± 0.34 mg drug, calculated as MTX free acid), which correspond to a dose of 19.8 ± 2.56 mg/kg.

To prevent hair interference with MN application, rats in cohort 2 were sedated using gas anaesthesia (2–4% isoflurane in oxygen), and the hair on the back was removed by using an electric clipper and depilatory cream (Boots Expert®, The Boots Company PLC, Nottingham, UK). On the next day, the animals were anaesthetised again, and two MTX NC-MN (total path size 1 cm^2^) was applied to the back of each animal with firm finger pressure. The MN were secured in place with Microfoam™ surgical tape (3 M, Bracknell, UK) and then an adhesive, vapour-permeable, thin film of Tegaderm™ (3 M, St Paul, Minnesota, USA). To further secure the MN in place, Kinesiology™ tape (ProWorks Corporation, Corvallis, USA) was then gently wrapped around the back and abdomen of each rat. The MN was applied for 24 h even though the MN dissolution time was much shorter because anaesthesia is needed to remove the MN arrays which cannot be applied more than once within 24 h, as per the Institutional Project Licence.

To evaluate MTX pharmacokinetics after oral and MTX NC-MN administration, blood samples with a maximum of 200 µL from each rat were collected in Eppendorf tubes (containing 10 µl heparin) at pre-defined time points (1, 2, 3, 6, 24, 48 and 72 h) through tail vein bleeding. These blood samples were kept at -80  °C until processing and analysis. Following the Project License restricting blood sampling volumes and times per day, five animals were bled at each time point, and a population pharmacokinetics approach was used to construct MTX pharmacokinetic profiles.

To evaluate MTX dermatokinetics, some rats (n ≥ 3) from each cohort, were culled at three-time points (2 h for MTX NC-MN cohort only, 24 and 72 h for both cohorts). Skin samples were harvested, processed and then analysed for MTX content. For rats in cohort 2, after cleaning with 1 ml PBS (pH 7.4) solution three times and wiping with a paper tissue, an approximately 0.5 cm^2^ skin biopsy sample from MN application sites and at a distance of 2.5 ± 0.5 cm away from the application sites were harvested using a disposable scalpel. We collected skin samples away from the application site to evaluate MTX distribution by the dermal circulation and the likelihood of spreading to the nearby areas. For rats in cohort 1, an approximately 0.5 cm^2^ skin sample from the back of each animal was also harvested. Subsequently, all skin samples were stored individually in 2 ml Eppendorf tubes and kept at -80  °C until processing and analysis.

#### 2.6.2 Blood samples processing procedures

The frozen blood samples were thawed. The freezing-thawing process allows for red blood cells (RBC) to be haemolysed, releasing MTX from the RBC into the plasma (Pegg and Diaper, 1988). After thawing, blood samples were centrifuged at 3000 x g at 4  °C for 15 min. The supernatant blood extracts were collected. To an aliquot of 100 µl of the supernatant blood extract, 300 µl of methanol were added (for blood protein precipitation) and vortexed for 15 min, then centrifuged at 14,000 x g for 15 min at 4  °C to extract MTX. The supernatant was then collected in a glass culture tube. Following this, the glass culture tubes were placed in a Zymark TurboVap® LV Evaporator Workstation (Lab & Life Science, Lab Equipment, UK) at 35  °C for 30 min for solvent evaporation. The residue was then reconstituted with 100 µl of DI water. This solution was then vortex mixed for 30 s and centrifuged at 14,000 × g for 15 min at room temperature. The supernatant was transferred into an Agilent® HPLC vial, and 10 µl was injected into the HPLC-MS system to measure MTX content using the validated method described below in the pharmaceutical analysis section.

#### 2.6.3 Skin samples processing

The biopsied skin samples were thawed and cut into small pieces using sharp scissors. Subsequently, 1 ml of methanol along with three stainless steel beads were added to each skin sample and then homogenised at 50 Hz for 10 min using TissueLyser LT (Qiagen, Ltd, Manchester, UK). The homogenised skin samples were then centrifuged at 14,000 ×*g* for 15 min before the supernatant was collected and transferred to a glass culture tube. The samples were then processed, as mentioned before in Section 2.6.2 and analysed for MTX content using the validated HPLC-MS method described below in the pharmaceutical analysis section.

### 2.7 Pharmaceutical analysis

#### 2.7.1 HPLC-UV method

This method was used to quantify MTX in samples collected from the *in vitro and ex vivo* studies. The analysis was carried out using Agilent 1220 Infinity compacted LC Series (Agilent Technologies UK Ltd., Stockport, UK) consisting of an Agilent degasser, binary pump, standard autosampler and UV Detector. The analytical method was adapted from (Begas et al., 2014) with minor modifications and validated as per the International Conference on Harmonisation (ICH) guidelines. The drug separation was achieved on Waters® Spherisorb ODS1 column (150 mm x 4.6 mm i.d. with 5 µm particle size) (Waters Corporation Milford, MA, USA) which was housed at 30 ± 1 °C. An isocratic mobile phase consisted of a mixture of ammonium acetate buffer 20 mM (pH 3.6): acetonitrile (90:10, v/v) was pumped at a flow rate of 1 ml/min. The drug was detected using a UV detector set up at a wavelength of 305 nm. The injection volume was 10 µl and the analysis run time was 6 min. Data acquiring, processing and storage were performed using Agilent ChemStation (B.04.02) Chromatography Data System.

#### 2.7.2 HPLC-MS method

This method was developed and validated as per ICH guidelines and then used to analyse MTX in samples collected from the *in vivo* study. The analysis was performed utilising again the same Waters® Spherisorb ODS1 column, which was coupled with Agilent 1260 Infinity-II LC series (Agilent Technologies, UK). The HPLC-MS consisted of a quaternary pump, an Agilent degasser, multi sampler, multicolumn thermostat and a single quadrupole mass spectrometer (MS) (API 6400, Agilent Technologies, UK). An isocratic mobile phase consisted of a mixture of water (with 0.1% v/v formic acid): acetonitrile (90:10 v/v) was pumped at a flow rate of 0.5 ml/min. The injection volume was 10 µl. The column temperature was maintained at 30 ± 1  °C. Drug quantification was achieved using the MS system, which was set up on ESI+ mode and a single ion monitoring (SIM) for analytes detection at *m/z* of 455.2 (equivalent to MTX Mw +1). Nitrogen was utilised as the source vapour and was kept at a pressure of 100 psi. The drying gas temperature and flow were 300  °C and 11 L/min, respectively. The nebuliser pressure was 15 psi, and the capillary voltage was established at 4 kV. The analysis run time was 10 min. Data acquiring, processing and storage were performed using Agilent Open LAB Chromatography Data System.

### 2.8 Statistical analysis

All results were expressed as means ± standard deviation (SD), n ≥3. These were calculated using Microsoft® Excel 2013 (Microsoft Corporation, Redmond, USA). Statistical analysis was carried out using GraphPad Prism® version 6 (GraphPad Software, San Diego, California, USA) followed by Tukey's multiple comparisons post-hoc test and *t*-test for two-group comparisons. A value of *p* < 0.05 was considered statistically significant.

## 3 Result and discussion

### 3.1 Characterisation of the MTX NC

In this study, the criteria were set to prepare MTX NC with a nano-size range (MTX NC), between 200 and 1000 nm. MTX NC with this particle size range offers two advantages. Firstly, they can be homogeneously embedded in the MN hydrophilic polymeric matrix (Nguyen and Banga, 2018). Secondly, they can be retained within the skin once delivered by the MN, allowing the drug to be released in a sustained manner (Zhu et al., 2013). MTX NC were produced using PVA 10 K as a stabiliser and acid-base neutralisation as a precipitation technique with sonication.

With respect to the NC stabiliser selection, PVA 10 K was chosen due to its advantages over other potential stabilisers. These including (i) being a biocompatible polymer with relatively small MW (9–10 kDa) so that it can be readily cleared from the body (Kamarul et al., 2014), (ii) it has a long history of safe use in fabricating various drug delivery systems (Ikada, 1995) and (iii) it is well-known for being insert and has no negative impact on the MN mechanical properties once the NC are incorporated into their shafts (Vora et al., 2018).

The preparation was chosen because it is simple, cost-effective and does not require use of toxic organic solvents. Importantly, the particle size of the NC can be controlled by adjusting the experimental conditions. MTX is a weak carboxylic acid with pKa values of 4.7 and 3.36 and mainly exists in two chemical forms; either as a disodium salt (MTX Na) or as MTX free acid (MTX) (dos Santos et al., 2017). While MTX Na is freely water-soluble because its two carboxylic groups are fully ionised, MTX as a free acid is poorly water soluble (0.01 mg/ml) (Kasim et al., 2004). Reducing the pH of the MTX Na solution below its pKa value 4.7 can lead to a formation of the poorly water-soluble MTX as crystals of various particle sizes depending on the experimental conditions (dos Santos et al., 2017). After a series of experiments to evaluate the effect of various experimental conditions such as the stabiliser, CA and MTX Na concentrations in their solutions, drug solution: stabiliser solution mixing ratio, drug titration speed and volume, sonication cycles, time and the amplitude on MTX NC properties by single-factor screening (data are not reported), MTX NC were produced, and its dispersion pH was 4.22.

#### 3.1.1 Particle size, PDI and zeta potential of MTX NC

Results showed that MTX NC was produced with mean particle size and PDI of 678 ± 15 nm, 0.248 ± 0.002, respectively, indicating the utilised method was useful in producing a homogenous monodisperse MTX NC within the desired particle size range. The zeta potential value was -31.3 ± 2.5 mV. The negative zeta potential value of MTX NC can be attributed to the partly ionised carboxylic acid groups in the MTX molecule, which occur at a pH between the two MTX pKa values of 4.71 and 3.36. This is in good agreement with previously published research (dos Santos et al., 2017). The high zeta potential value, in addition to the presence of PVA 10 K as a stabiliser, should be sufficient to avoid MTX NC aggregation and promoting its physical stability (Wang et al., 2013).

#### 3.1.2 SEM spectroscopy

**Fig. 2:**
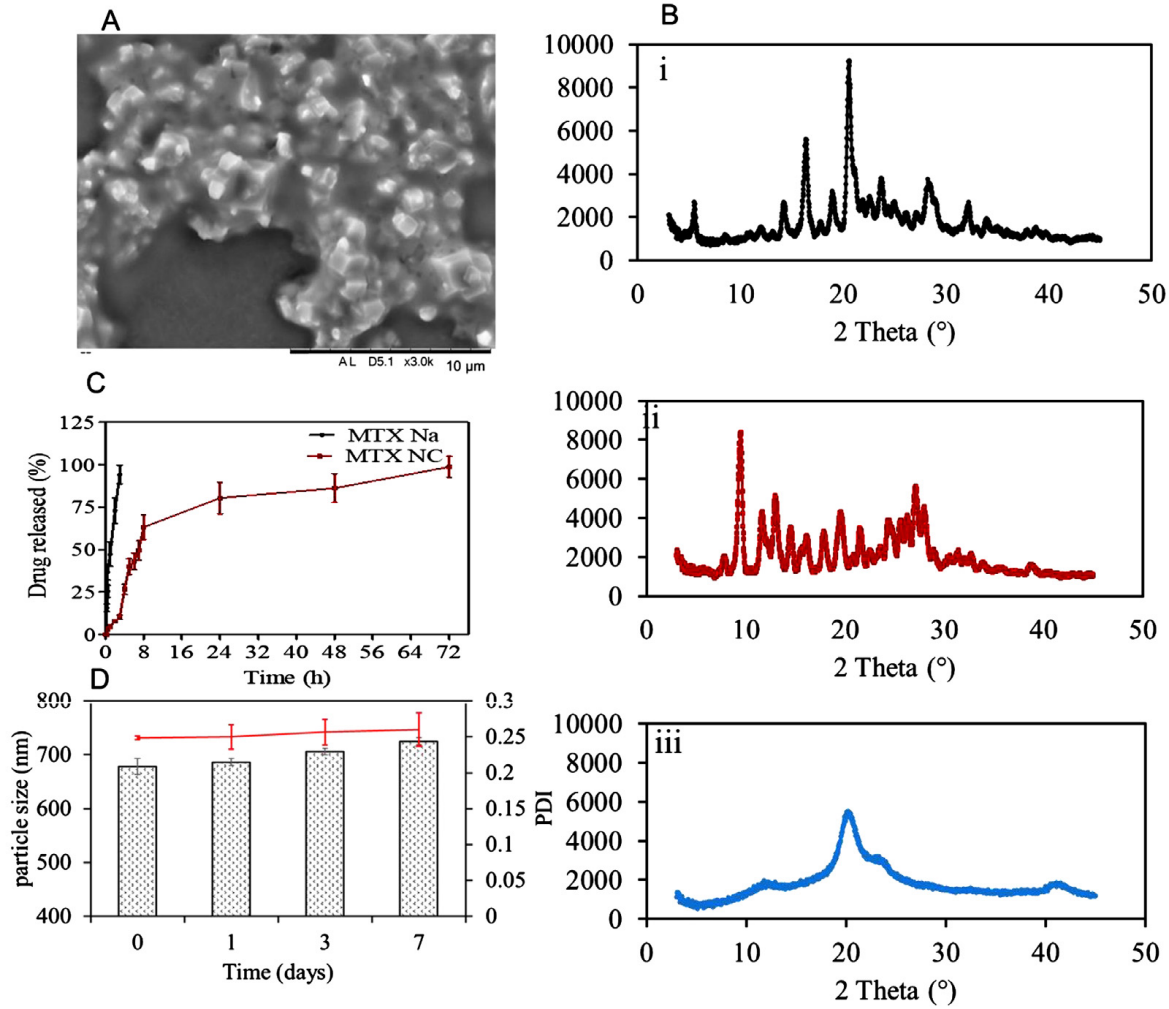
(A) Representative SEM image of MTX NC. The black scale bar represents a length of 10 µm; (B) XRD diffractograms of i) MTX Na; ii) MTX NC and iii) PVA 10 K; (C) *In vitro* cumulative release profiles of MTX (presented as % of the applied dose) from MTX NC suspension and aqueous MTX Na aqueous solution after being loaded in the Spectra-Por®, 12,000–14,000 MWCO dialysis bag and immersed in 100 ml of PBS (pH 7.4) at 37 ± 1  °C. Data are reported as means ± SD, n = 3; D) MTX NC particle size and PDI over seven days period. Data are reported as means ± SD, n = 3.

To get an insight into the morphology of the NC, samples of the fabricated MTX NC were visualised using SEM. The micrographs (Fig. 2A) showed that the produced MTX NC appeared as crystals in approximately octahedron shape embedded in a pool of amorphous materials which is likely to be the stabiliser- PVA 10 K. This is again in good agreement with previous studies, which have indicated that the polymeric stabiliser forms a thin film to coat the drug nanocrystals (Vora et al., 2018; Yu et al., 2018). The observed particle size of MTX NC was < 1000 nm, which again consistent with the results obtained from the particle size analyser.

#### 3.1.3 HPLC-UV method validation

For the *in vivo* and *ex vivo* studies, MTX was quantified using the validated HPLC-UV method. MTX eluted at 4.91 min with no interference with any impurities from the analysed matrices. The detector response was linear in the range of 0.28- 10 µg/ml (R^2^ > 0.9999). The slope was 126.03. The method showed excellent intra-day and inter-day accuracies (RE%< 0.66% and 2.35%, respectively) and precisions (RSD% were <3.34 and 4.14%, respectively) (n = 6). The limit of detection (LoD) was 0.07 µg/ml, and the limit of quantification (LoQ) was 0.28 µg/ml and the percentage of MTX extraction recovery from the skin samples was in the range of 91.88 ± 2.14%.

#### 3.1.4 Drug content

To evaluate the effect of NC preparation process and conditions on the MTX stability and integrity, the drug content in the fabricated MTX NC was determined. The amount of MTX recovered from MTX NC was 176.4 ± 2.5 mg which equates to a percentage recovery of 96.9 ± 1.41%, indicating that the MTX NC preparation method and conditions did not have any negative impact on MTX stability and integrity.

#### 3.1.5 XRD analysis

XRD analysis was carried out to confirm MTX NC formation by comparing its crystalline structure with that from its parent compound, *i.e.* MTX Na. XRD analysis of the stabiliser PVA 10 K was also performed to investigate the potential for interference. Fig. 2B shows the XRD patterns of the three materials. As can be seen from XRD diffractograms Fig. 2B(ii), MTX NC exhibited crystalline peaks at 2*θ* values of 10 and 27^∘^, which are the characteristic peaks for MTX (free acid). This was different from MTX Na XRD diffractograms, which showed different typical peaks at 2*θ* values of 17 and 21° (Fig. 2B(i)). This is consistent with the results obtained from SEM and particle size analyser studies. It also comes in good agreement with a previous study (Huang et al., 2014). The XRD diffractograms of PVA 10 K (Fig. 2B(iii)) showed a crystalline peak at 2*θ* value of 19° which also appeared as small peak in XRD diffractograms of MTX NC. This can be ascribed to the PVA 10 K being used as a stabiliser at a low concentration (2% w/w). These results suggest that the described preparation method was useful in producing MTX NC with the desired properties.

#### 3.1.6 In vitro drug release and release kinetics

*In vitro* drug release was performed to evaluate the ability of MTX NC to control MTX release once deposited in the skin tissue by the MN. Taking into consideration that MTX solubility is a pH-dependent (dos Santos et al., 2017) and that the pH of the skin interstitial fluid in the skin is around 7.4, PBS 10 mM (pH 7.4) was selected as a release medium to mimic the skin interstitial fluid. As a control, *in vitro* drug release from MTX Na aqueous solution was also evaluated. The *in vitro* release profiles from both MTX NC and MTX Na are illustrated in Fig. 2C. As can be seen from the graph, almost 100% of MTX Na was released within 3 h from immersion in the release medium. In contrast, MTX NC exhibited a typical sustained release profile extended up to 72 h. No burst release was observed, as only 12 ± 1.2% of the loaded drug was released within the first 3 h. It is worth noting that MTX NC has a biphasic release profile. In the first phase, MTX was released at a relatively fast rate; thus, around 63.2 ± 7.3% of the loaded drug was released within the first 8 h. In the second phase, the drug was released at a much slower rate. Thus, the remaining drug (~37%) was released over the next 64 h. This can be ascribed to the fact that MTX NC is poorly water-soluble. Thus, it has a low dissolution rate in the PBS in comparison with MTX Na, which is freely water soluble. Accordingly, its dissolution rate was significantly higher in comparison (*p* < 0.05). Taking into consideration that the volume of interstitial fluid in the skin is much smaller than the volume of release medium utilised in this study, we would expect that MTX NC will liberate the drug at a much slower rate, thus sustaining its release, even for longer than 72 h.

To investigate the kinetic modelling and release mechanism of MTX from its NC, the resultant *in vitro* release profile of MTX NC was fitted to several release kinetic models, specifically, first-order, zero-order, Korsmeyer- Peppas and Higuchi models. Calculating R^2^ value of each model, showed the following values 0.9522, 0.4335, 0.8724 and 0.8811, respectively, indicating that the first-order model would best describe the MTX release profile from its MTX NC. Accordingly, the MTX release rate from its MTX NC is dependent upon MTX loading in the tested formulation (Permana et al., 2019).

#### 3.1.7 Stability of MTX NC

Short-term physical stability of MTX NC was carried out to ensure that MTX NC does not aggregate upon storage. Thus, MTX NC particle size, PDI and zeta potential were measured at pre-determined time intervals over seven days. Results revealed that the particle size and PDI of MTX NC exhibited slight but not significant (*p* > 0.05) increase in the particle size and PDI over the seven days (Fig. 2D). A similar trend was observed for zeta potential, indicating that MTX NC was stable and continued as a monodisperse system. No sign of any aggregation was observed over the study period.

### 3.2 Characterisation of dissolving bilayer MTX NC-MN

In general, traditional dissolving MN designs are meant to provide a simple, minimally invasive method to deliver a low dose of potent hydrophilic drug to the skin. The amount of drug delivered usually depends on the amount of drug embedded within the MN shafts in addition to a drug diffusion from baseplate after MN insertion. In our study, a poorly water-soluble MTX NC was used. Thus, the only drug loaded into the MN shafts part that is inserted into the skin would likely to be delivered, but not from the baseplate (Mc Crudden et al., 2018). Therefore, to avoid drug wastage, bilayer MN arrays were fabricated in which MTX NC was loaded only into the MN shafts by the two-step manufacturing process.

Concerning drug loading, taking into consideration that our drug delivery system is designed for sustained release, we aimed to prepare MN with the maximum possible loading and satisfactory mechanical and insertion properties. Thus, a reasonable patch size can be used to deliver the required dose of MTX.

Looking into the polymeric materials that were used in MN fabrication, the criteria was set to select a blend of biocompatible polymers with a long history of safe use that would form a mechanically robust MN and their MW to be < 60 kDa so that once deposited in the skin, and they can be cleared from the body readily. In the literature, many water-soluble, biocompatible polymers have been used to prepare to dissolve MN, including PVA (Vora et al., 2018), PVP (Ramöller et al., 2019) and Gantrez® copolymers (Hamdan et al., 2018). Out of these polymers, a blend of PVP (MW = 58 kDa) and PVA 50 K (MW = 31–50 kDa) were selected for MN fabrication. Because PVP and PVA 50 K are biocompatible polymers with MW <60 kDa, thus, they can be eliminated through the kidneys (Ikada, 1995; Hespe et al., 1977). The elimination rates of PVA polymers depend primarily on the molecular weight of the polymer, the lower the MW size, the faster the elimination (Ikada, 1995). The polymeric blend composition to produce robust MN arrays was select after a series of experiments investigating the effect of various mixing ratios of the two polymeric hydrogels on MN mechanical properties (data were not shown). The best composition of the polymeric blend was found to be a hydrogel consisting of PVP and PVA 50 K added at 20% and 15% w/w, respectively.

**Fig. 3:**
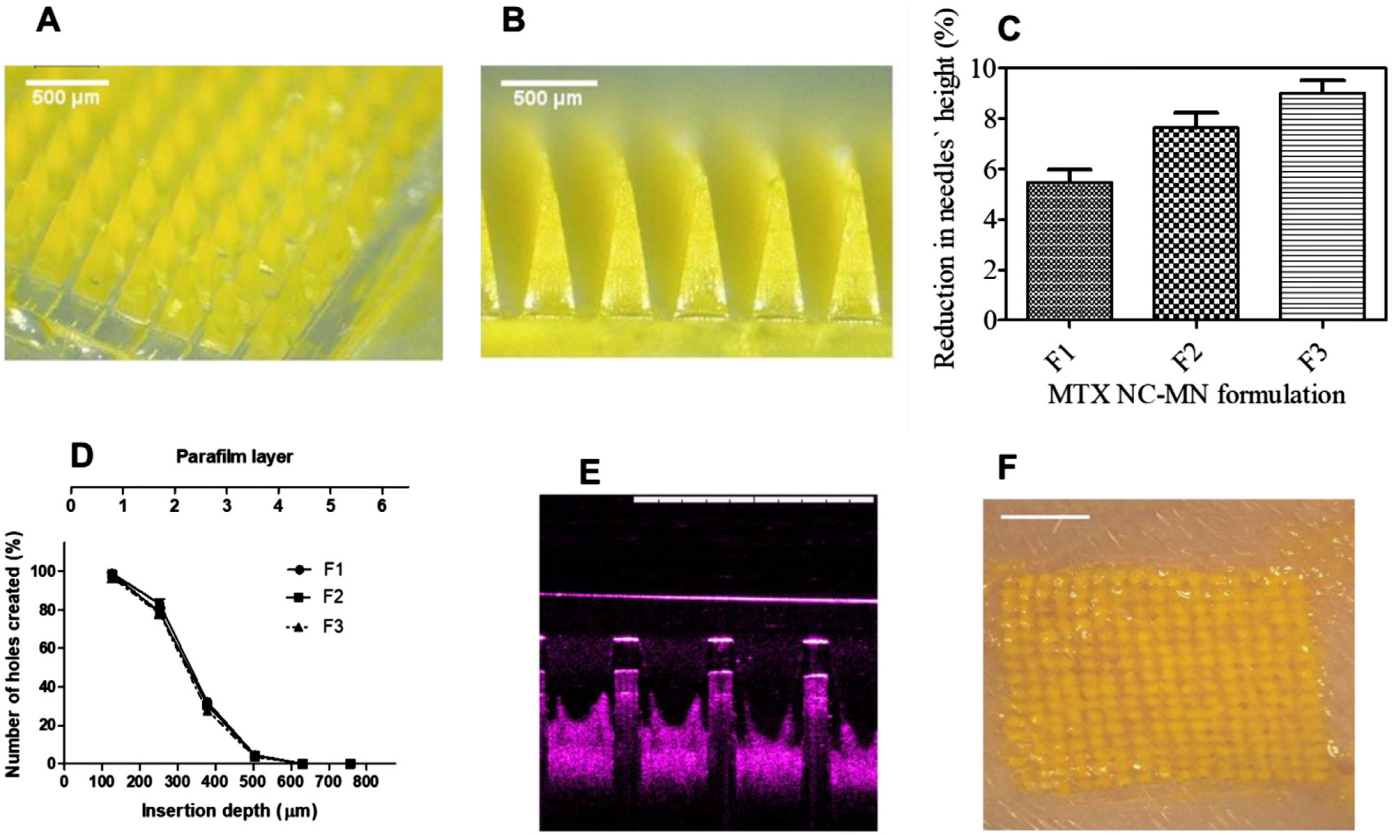
Representative light microscopic images for (A) panoramic view; (B) side view of MTX NC-MN array prepared from MTX NC-loaded hydrogel formulations (F3) showing MTX NC loaded in the MN needles only; (C) Comparison of Comparison of the percentage of height reduction of MN needles formulations containing the percentage of MN shaft`s height reduction of MTX NC-MN prepared from various MTX NC-loaded hydrogel formulations (F1, F2 and F3) after applying a compression force of 32 N per MN array vertically against artificial skin-simulant membrane consisting of a stack of 8-layer of Parafilm M® (mean ± SD, n = 3), (D) Number of Parafilm M® layers inserted, approximate insertion depths and percentage of holes created by MN shafts in each Parafilm M® layer using an insertion force of 32 N/MN array using the Texture Analyser for MTX NC-MN arrays prepared from various MTX NC-loaded hydrogel formulations (F1, F2 and F3) (mean ± SD, n = 3); (E) A representative OCT image of MTX NC-MN prepared from MTX NC-loaded hydrogel formulation (F3) following insertion into excised full-thickness neonatal porcine skin. The white scale bar represents a length of 1 mm; (F) Representative light microscopic image of the surface of full-thickness porcine skin following insertion the MTX NC-MN prepared from MTX NC-loaded hydrogel formulation (F3) for 20 min and then removing the baseplate. The white scale bar represents a length of 2 mm.

With respect of MTX NC- MN array fabrication, four MTX NC-loaded hydrogel formulations (F1, F2, F3 and F4) were assessed. MN arrays were successfully fabricated from the first three formulations. Representative light microscope images for MTX NC-MN prepared from F3 are depicted in Fig. 3A and 3B. The light microscope images clearly show that bilayer MN arrays were formed with the drug being loaded mainly in the MN shafts. No drug was observed in the baseplate. Each MN array had a total surface area of 0.5 cm^2^ and consisted of a 16 × 16 array, which equates to 256 needles of a square-based pyramid with side-length of 300 µm at the baseplate and needle-needle interspacing of 100 µm. These dimensions are consistent with the theoretical values of the PDMS mould. However, the needles` height was ≈800 µm, which is slightly less than the theoretical needle height in the PDMS mould. This can be ascribed to the polymeric matrix shrinking slightly upon drying. It is worth noting that F4 produced MN with brittle shafts that were broken upon removal from the MN moulds. This can possibly be attributed to the high drug loading, which was around 75% w/w. Therefore, F4 was discarded.

#### 3.2.1 Drug content

The amount of drug-loaded into the MN shafts has a significant impact on the dose delivered into the skin (Vora et al., 2018; Mc Crudden et al., 2018; Permana et al., 2019). Therefore, it was essential to determine MTX NC-MN drug content. This was calculated using the volume of needles per MN array and the density and drug concentration of needle-free, MTX NC-loaded patch. The density of the needle-free, MTX NC-loaded patch was found to be 1.22 ± 0.11 mg/mm^3^. Hence, the amount of MTX (calculated as MTX free acid) in each MN array prepared from MTX NC-loaded formulations F1, F2 and F3 would be 0.41, 1.1 and 2.48 mg per 16 × 16 MN array, respectively. These equate to 5.2, 11.4 and 31.1% w/w of the needles` dry weight, respectively. These present dramatic increases (*p* < 0.001) in the amount of MTX loaded into the MN shafts in comparison with the amount of MTX Na loaded in the hyaluronic acid-based MN that has been previously reported which was only 13.8 µg/MN array (Du et al., 2019).

#### 3.2.2 Effect of the MN polymers and fabrication process on MTX NC properties

The stability of MTX NC within the MN is very crucial to maintain the ability of the drug to be released in a controlled and consistent fashion (Mc Crudden et al., 2018; Permana et al., 2019). The particle size of the MTX NC should not be modified after being combined with the polymeric hydrogel or after drying form the MN arrays. Consequently, the particle size, PDI and zeta potential of MTX NC were evaluated before and after the MN fabrication process. Results revealed that the particle size, PDI and zeta potential of the MTX NC recovered from the finalised MTX NC-MN (has the highest possible drug loading) were in the region of 689 ± 13 nm, 0.245 ± 0.006, and -30.22 ± 0.46 mV, respectively. These values were not significantly different (*p* > 0.05) from the previously reported values for MTX NC before being embedded in the MN polymeric matrix, indicating that the MN array matrix and preparation process has no negative impact on the MTX NC physical stability and therefore continued to be a monodisperse system.

#### 3.2.3 Mechanical and insertion properties of the MN

The capability of the MN to be efficiently inserted into the skin is critical to its use, as the *stratum corneum* must be penetrated by the MN to exert its effects. Incorporation of drug substances into the polymeric matrix of the MN can create either a weakening or strengthening impact on the MN mechanical properties (Hamdan et al., 2018; Hespe et al., 1977). Therefore, the mechanical and insertion properties must be determined as an integral part of initial formulation studies for dissolving MN (Larrañeta et al., 2016). The percentage of height reductions of needles on the various MN arrays were determined after applying a compression force of 32 N/array, equitable to manual compression force likely to be applied onto the MN upon insertion into the skin (Larrañeta et al., 2014). Results revealed that the MN drug content affects their mechanical properties, the higher the drug content, the higher the percentage of the needles` height reduction. However, MN arrays prepared from the three formulations had a percentage of height reduction of less than 10% (Fig. 3C), and none of the needles was broken or shattered, indicating that the fabricated MN will be strong enough to be inserted into the skin (Larrañeta et al., 2016).

Concerning the insertion ability, it was found that the MN arrays prepared from three formulations were able to penetrate four layers of the stack of 8 layers of Parafilm M®, which has been shown to be a good alternative model for human skin for MN insertion studies (Larrañeta et al., 2014). As the mean thickness of each layer of Parafilm® is approximately 126 μm (Larrañeta et al., 2014), the MN were inserted down to 504 μm which equates to 63% of the needles` height being successfully inserted (Fig. 3D). Statistical analysis showed that there was no significant different (*p* > 0.05) in the insertion ability of any of the tested MN arrays. We aimed to prepare MN with the maximum possible loading and satisfactory mechanical and insertion properties. It was observed that MTX NC-MN prepared from formulation F3 has the highest drug loading and exhibited a satisfactory mechanical strength (Mc Crudden et al., 2018; Permana et al., 2019) and inserted well in the Parafilm M®. Therefore, it was selected as the “finalised MTX NC-MN” and were subjected to further testing.

For MN skin penetration study, a non-invasive optical imaging technique (OCT) was used to acquire real-time images of the insertion of the finalised MTX NC-MN into the neonatal full-thickness porcine skin (*ex vivo*). The two-dimensional side-view image (Fig. 3E) demonstrated that MTX NC-MN possessed the capability to be inserted into neonatal porcine skin, reaching insertion depth of approximately 485 ± 35 µm, which equates to 60.6 ± 4.1% of the needles` total length. The insertion was also confirmed by light microscope visualising (Fig. 3F), which clearly showed the holes in the skin created by MN shafts and filled with the drug particulates (yellow colour) following the MN application for 20 min. It is worth mentioning that the MN insertion into porcine skin was slightly lower than that observed in the Parafilm M® layers. However, statistically, there was no significant difference (*p* > 0.05) exerted between the MN insertion capabilities into either the Parafilm M® or the full-thickness neonatal porcine skin. These findings are consistent with results of other studies, which have been previously reported (Ramöller et al., 2019; Vora et al., 2018; Larrañeta et al., 2014). Taking into consideration that porcine skin is a good model for human skin (Larrañeta et al., 2016; Summerfield et al., 2015).These results demonstrate that our MTX NC-MN would be able to penetrate the outermost layer of the skin in human and reach the dermis, thus confirming their potential for MTX intradermal delivery.

#### 3.2.4 Ex vivo dissolution kinetics on MTX NC-MN

**Fig. 4:**
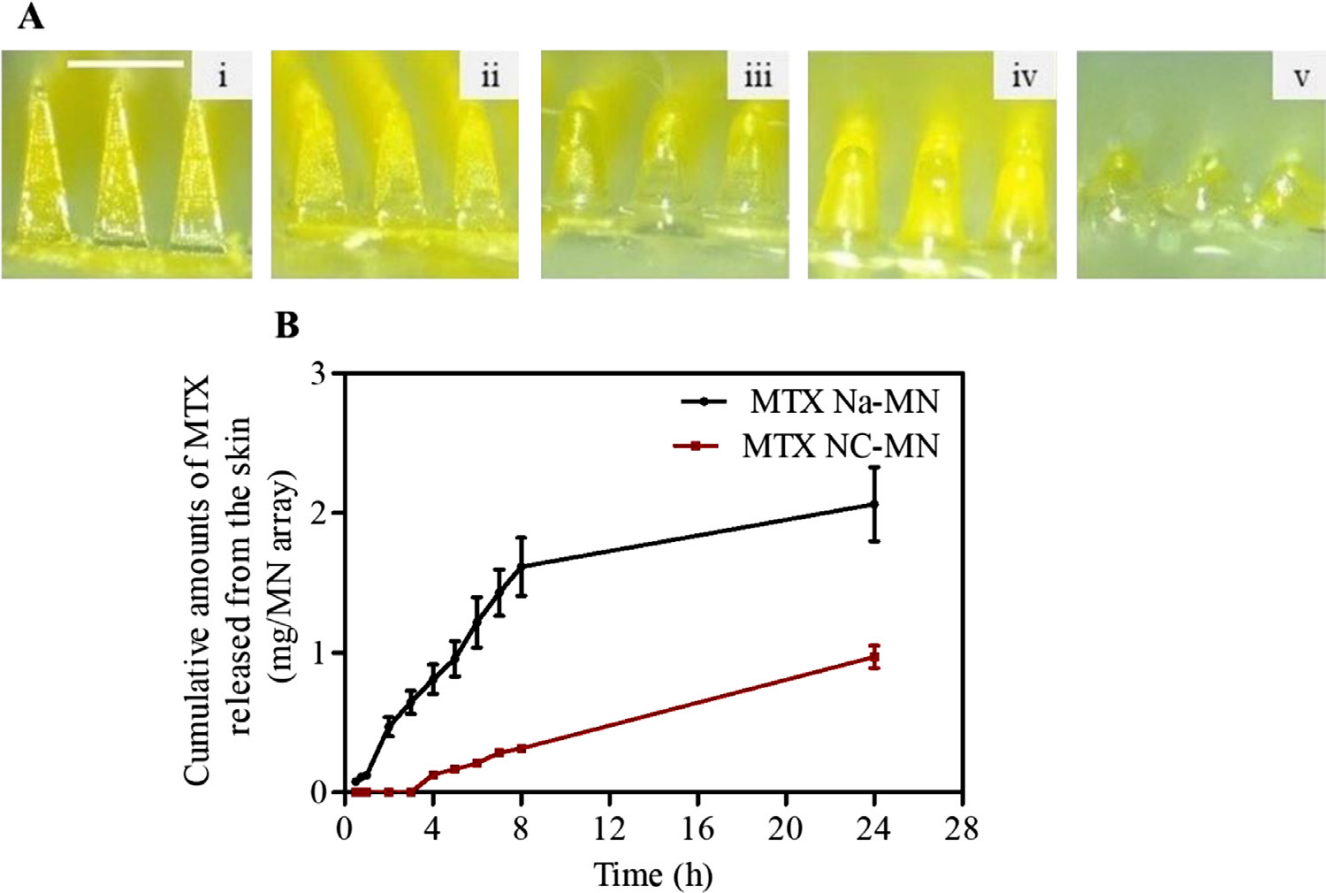
(A) Representative light microscope images of MTX NC-MN prepared from MTX NC loaded hydrogel formulation F3 after insertion into full-thickness neonatal porcine skin for the following periods: (i) 0 min, (ii) 5 min, (iii) 10 min, (iv) 15 min and (v) 20 min. The white scale bar on the upper lift side of the images represents a length of 0.5 mm; B) Ex vivo MTX release profiles from an excised full-thickness neonatal porcine skin to PBS solution housed at 37 ± 1  °C following MTX Na-MN and MTX NC-MN application. Data are reported as means ± S.D., n = 3.

Moving further with the finalised MTX NC-MN prepared from formulation F3 (Fig. 4 (i)), dissolution kinetic study was performed to estimate the MN wearing time by which MN can dissolve and release their MTX NC cargo in the skin. The short wearing time is better and the more convenient, consequently, better patient compliance (Larrañeta et al., 2016; Ita, 2015). Results revealed that, within 5 min from inserting the MN into the skin, the needles started to dissolve, a noticeable reduction in the height of the needle was observed (Fig. 4 A(ii)). The dissolution continued over the period 10 and 15 min as can be seen in Fig. 4 A(iii) and 4A(iv), respectively. Complete needle dissolution was achieved after 20 min (Fig. 4A(v)), indicating a relatively short wearing time is required for these MN dissolve and release their MTX NC cargo in the skin. It is essential aspect of such a drug delivery system in the clinical application as it improves its acceptability from the patients.

#### 3.2.5 Ex vivo MTX dermatokinetic and release studies

These studies aimed to evaluate the ability of MTX NC to retain the drug at the application site in the skin and sustain its release following its intradermal delivery by the MN. These were achieved by studying the kinetic profiles of MTX in the skin (namely deposition, distribution and then its release from the skin) following MN insertion and depositing their payload in the skin. For comparison, MTX dermatokinetics and release following MTX Na-MN application (as a control) were also evaluated.

Concerning the release studies, Fig. 4B presents the MTX release profiles from the excised full-thickness porcine skin to the PBS solution following applying the finalised MTX NC-MN (prepared from formulation F3) and the control MTX Na-MN. When the sodium version of MTX (MTX Na-MN) (containing around 2.48 mg MTX Na/MN array) was applied into the skin, the drug was detected in the receptor solution within the first 0.5 h (the first sampling point) from MN application. After 3 h, approximately 0.65 ± 0.08 mg (around 25% of the loaded dose) released from the skin and the drug continued its release from MN at a fast rate so that after 8 h, most of the applied dose (1.61 ± 0.21 mg, equates to approximately 65% of the applied dose) was released from the skin. After 24 h, 2.06 ± 0.26 mg (Almost 85% of the loaded MTX Na) was released from the skin and accumulated in the receptor solution of Franz-diffusion cells.

In contrast, after MTX NC-MN (containing 2.48 mg MTX/MN array) application, no drug was detected in the receptor solution within the first 3 h from MN application. After 8 h, only 0.31 ± 0.03 mg of MTX (around 12.5% of the loaded dose) released from the skin. MTX release continued at a slow rate until the end of the study period (24 h), by which less than 0.97 ± 0.08 mg of MTX (around 39% of the loaded dose) was released in the receptor solution. The cumulative amount of MTX released from the MTX NC-MN was substantially (*p* < 0.05) less than (approximately 46%) the amount of MTX released from MTX Na-MN, indicating that MTX NC-MN was able to deliver MTX NC into the skin and sustain its release. Notably, the amounts of MTX which were delivered intradermally by both MTX Na-MN or the MTX NC-MN were substantially higher than those reported to be delivered by passive permeation using conventional MTX Na topical formulations (Prasad and Koul, 2012; Nguyen and Banga, 2018; Weinstein et al., 1989).

**Fig. 5:**
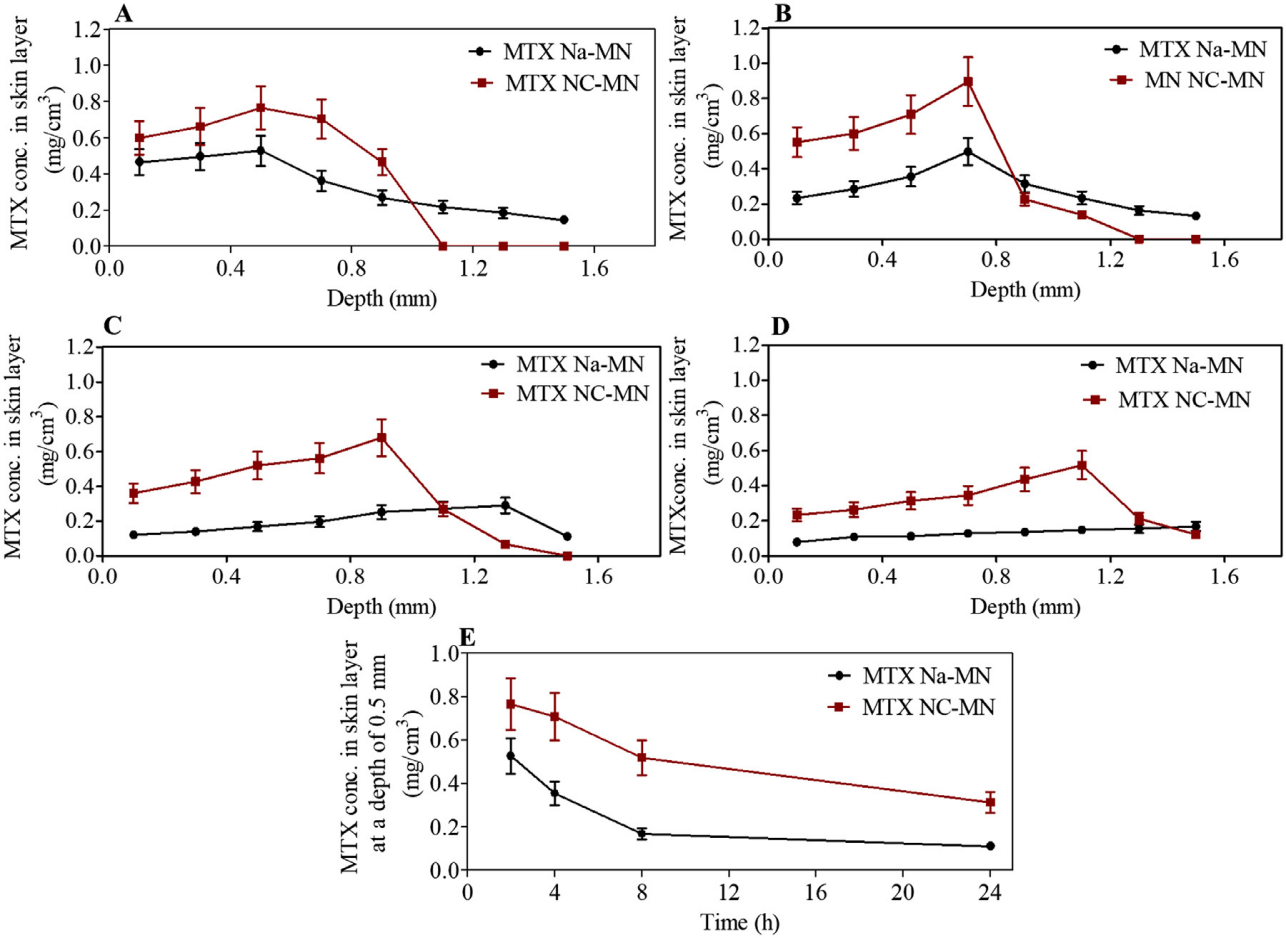
MTX concentrations (mg/cm3) in various layers of neonatal full-thickness porcine skin (Ex vivo), following the application of the MTX Na-MN and MTX NC-MN array after (A) 2 h, (B) 4 h, (C) 8 h and (D) 24 h., E) A representative of MTX concentrations (mg/cm3) changes *vs* time (h) in the skin layer of neonatal full-thickness porcine skin (Ex vivo) at depth 0.5 mm following the application of the MTX Na-MN and MTX NC-MN arrays. Data are reported as means ± S.D., n = 3.

For MTX deposition and distribution in the skin, Fig. 5(A-D) depict the amount of MTX (mg) detected per cm^3^ in a range different depths of skin after four application times, namely, 2, 4, 8, and 24 h from applying MTX NC-MN and MTX Na-MN (the control) into the skin. As can be observed, the MN were able to deliver the MTX NC, which in turn, was able retain the drug at the application site in the skin. Thus, even after 24 h from MN application, most of the drug was still retained in the upper layers of the skin (where the MN shafts reached). However, the MN which contain MTX Na (MTX Na-MN) were able to deliver the drug into the skin, which then distributed quickly in all skin layers even within 2 h from MN application. After 24 h, most of the delivered dose was cleared from the skin. For example, from Fig. 5A, it can be clearly observed that, after applying MTX NC-MN for 2 h, the highest MTX concentration was found mainly in skin layers at a depth of 0.1 and 0.5 mm, where the MN was inserted. These results are consistent with results of the insertion studies reported in the previous section. At these depths, MTX concentrations were 0.61 ± 0.09 and 0.77 ± 0.11 mg/cm^3^, respectively. At deeper depths (0.9 and 1.1 mm), no drug was detected in the skin sections. In contrast, MTX Na-MN had no control and allowed MTX Na to go deeper in the skin beyond 1.1 mm even within 2 h from MN application. The MTX concentrations in skin layers at a depth of 0.1 and 0.5 mm were 0.47 ± 0.07 and 0.53 ± 0.08 mg/cm^3^. These concentrations are significantly (*p* < 0.05) lower than those reported from MTX NC-MN. Similar drug distribution patterns were observed at the other time points (4, 8 and 24 h), which are presented in Fig. 5B-D. Interestingly, in Fig 5D, it can be observed that even after applying MTX NC-MN for 24 h, MTX concentrations in the skin layers at depth of 0.1 and 0.5 mm continued to be relatively high and they were 0.21 ± 0.04 and 0.31 ± 0.05 mg/cm^3^, respectively. These were significantly (*p* < 0.001) higher than MTX concentrations in the skin at the same insertion depths (those were 0.11 ± 0.02 and 0.11 ± 0.02 mg/cm^3^, respectively) obtained after applying MTX Na-MN. Furthermore, by comparing the MTX concentration changes over 24 h following MN application (either MTX NC-MN or MTX Na-MN (the control)) at a certain depth (*e.g.* 0.5 mm) (Fig. 5E), it can be clearly seen that MTX NC were able to retain a high proportion of the delivered dose at the application site. Even 24 h from applying MTX NC-MN, drug concentration at 0.5 mm was maintained above 0.32 ± 0.05 mg/cm^3^, representing approximately 40% of that measured at 2 h from MN application at the same depth. In contrast, the drug concentration at 0.5 mm quickly declined to reach 0.11 ± 0.02 mg/cm^3^ after applying the MTX Na-MN (the control).

By comparing the cumulative amounts of MTX retained in the skin after applying both MTX NC- MN and MTX Na-MN, we found that substantial amounts of the applied dose were retained in the skin after applying MTX NC-MN, in comparison with the control (MTX Na-MN). For example, the total amounts of MTX retained in the skin after 2 h from applying MTX NC-MN were in the magnitude of 0.58 mg/cm^3^. It was around 1.2-fold higher than the total amounts of MTX (0.47 mg/cm^3^) retained in the skin after applying MTX Na-MN. Interestingly, after 24 h from MTX NC-MN application, the total amounts of MTX that retained in the skin were still high (0.453 mg/cm^3^) and equate to 78% of the total amounts of MTX that retained after 2 h form applying the same MN. However, this is substantially (*p* <0.001) (2.5-fold) higher than the total amounts of MTX retained in the skin (0.182 mg/cm^3^) after applying MTX Na-MN.

These results are consistent with the *ex vivo* and *in vitro* release studies results, indicating that the fabricated MTX NC-MN was able to deliver substantial amounts of the loaded MTX NC into the skin, which in turn, formed a drug depot in the skin that released the drug in a sustained manner. Our findings are in good agreement with previously published research which showed that MN loaded with NC bigger than 200 nm in size were able to deliver the drugs NC into the skin, after which they act as depots releasing drugs in a sustained manner (Vora et al., 2018; Mc Crudden et al., 2018; Abdelghany et al., 2019).

### 3.3 *In vivo* pharmacokinetics and dermatokinetics of MTX

The results from the *in vitro* and *ex vivo* studies revealed that MTX NC- MN was able to deliver MTX NC into the skin and provided a sustained drug release. However, these findings need to be ensured in an *in vivo* context. For that reason, the *in vivo* study was performed, to appreciate the performance of our novel drug delivery system, MTX pharmacokinetics and dermatokinetics was assessed after applying MTX NC-MN and compared with MTX Na that is administered orally (the commonly used administration route in therapy). This study was carried out using healthy female Sprague Dawley rats.

#### 3.3.1 HPLC-MS method validation

In the *in vivo* studies, MTX was quantified using the validated HPLC-MS method. MTX eluted at 6.47 min with no interference with any impurities from the analysed matrices. The MS detector response was linear in the range of 0.026- 5 µg/ml (R^2^ > 0.999). The slope was 2 × 10^6,^ and the intercept of the line was 1787.7. The method showed excellent intra-day and inter-day accuracies (RE%< 1.96% and 2.22%, respectively) and precisions (RSD% were <4.27 and 6.51%, respectively) (n = 6). The limit of detection (LoD) was 0.007 µg/ml, and the limit of quantification (LoQ) was 0.026 µg/ml. The percentage of MTX extraction recovery from blood and skin samples were in the range of 91.37 ± 2.94% and 91.88 ± 2.14%, respectively.

#### 3.3.2 MTX pharmacokinetics in blood

**Fig. 6:**
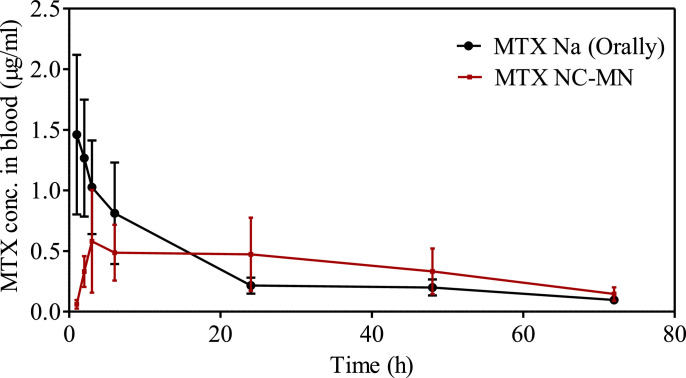
MTX pharmacokinetic profiles in blood after administrating MTX Na solution by oral gavage and MTX NC-MN application to healthy female Sprague Dawley rats. Data is reported as means ± S.D., (n = 5).

MTX pharmacokinetic profiles in blood following MTX NC-MN application and administrating MTX Na solution by oral gavage are presented in Fig. 6. MTX pharmacokinetic parameters were calculated and reported in Table 2. As can be seen from the graph, MTX maximum blood concentration (C_max_) showed a different pattern depending on the drug delivery system used. Orally administered MTX Na solution promoted a quick drug absorption with a C_max_ of 1.46 ± 0.65 µg/ml after 1 h. The drug concentration then declined rapidly to reach 0.81 ± 0.42 µg/ml after 6 h. After that, it continued to decrease gradually to reach 0.22 ± 0.06 µg/ml at 24 h. These results are in good agreement with other studies that have been previously reported in terms of T_max_ and C_max_ (Park et al., 2006; Kuroda et al., 2001).

**Table 2:** Pharmacokinetic parameters of MTX following MTX Na administration by oral gavage and after applying MTX NC-MN to healthy female Sprague Dawley rats. Data are reported as means ± S.D., n = 5.

Parameters	MTX Oral	MTX NC-MN
**Dose (mg/kg)**	2.5 ± 0.15	19.8 ± 2.56
**C_max_ (µg/ml)**	1.46 ± 0.65	0.58 ± 0.42
**T_max_ (h)**	1	3
**AUC_0-t_ (µg/ml.h)**	23.76 ± 6.31	26.29 ± 5.31
**AUC_0-INF_ (µg/ml.h)**	26.37 ± 7.35	32.22 ± 5.73
**Apparent t_1/2_ (h)**	18.86	28.24

In contrast, in the rats which received MTX NC-MN, the MTX peak concentration in blood (C_max_ = 0.58 ± 0.42 µg/ml), was (40% of C_max_ in the oral group) substantially (*p* < 0.05) lower than that observed in the oral group. Additionally, T_max_ = 3 h in the MN group was significantly (*p* < 0.001) longer than that in the oral group. However, the area under the curve (AUC) was very comparable between oral and MTX NC-MN (No significant difference, *p* > 0.05).

#### 3.3.3 MTX dermatokinetics

Since the primary target for MTX NC-MN application is to treat psoriasis, it would be very informative to conduct a dermatokinetic study to measure MTX concentrations (reported as µg/g) in the skin after MTX Na oral administration (the most common administration route) and after applying our novel MTX NC-MN. To get an insight into how drug defuses from the NC at the application site to the surrounding areas by dermal microcirculation, we measured MTX concentrations at both the MN application site and at nearby areas at 2.5 ± 0.5 cm away from it in the MN group. Results are summarised in Table 3.

**Table 3:** MTX concentration in the skin after administrating MTX Na by oral gavage and in the skin at the application site and 2.5 ± 0.5 cm away from the application site after applying MTX NC-MN. Data are reported as means ± S.D., n = 5.

Time (h)	MTX concentration in the skin (µg/g)		
	MTX Oral	MTX NC-MN	
		MN application site	2.5 ± 0.5 cm away from the MN application site
**2**	N/A	624.51 ± 405.97	166.32 ± 136.13
**24**	0.942 ± 0.59	312.70 ± 161.95	45.69 ± 24.52
**72**	0.172± 0.09	78.27 ± 30.02	20.12 ± 8.88

In the oral group, after 24 h from drug administration, MTX concentrations in the skin was 0.942 ± 0.59 µg/g. This was rapidly declined to reach 0.172 ± 0.09 µg/g (equates to 18% of the MTX concentrations after 24 h) at 72 h. In contrast, in the MN group, after 2 h from MN application, MTX was detected in the skin at both; the application site and in the nearby areas, and its concentrations were substantially (*p* < 0.001) higher than that reported in the oral group, and they were in the magnitude of 624.51 ± 405.97 µg/g and 166.32 ± 136.13 µg/g, respectively. It suggests that the skin microcirculation helped in distributing the drug to the nearby area, thus no need to apply use MN that cover the whole psoriatic patch. Instead, one MN can be applied in the cere or nearby of the affected area. It is very important in the clinical application.

After 24 h, MTX concentrations at the application site decreased only by 50% to reach 312.70 ± 161.95 µg/g, which is still 332-fold higher than MTX concentrations measured at the same period in the oral group. Interestingly, even after 72 h from MN application, MTX concentrations at the application site and the nearby areas were decreased gradually to reach78.27 ± 30.02 µg/g and 20.126 ± 8.88 µg/g, respectively, which but remained substantially higher than that reported in the oral group. These findings are consistent with the results obtained from the *in vitro* and *ex vivo* studies reported in the previous sections and give further support to our hypothesis that the incorporation of MTX NC into MN would be able to deliver MTX NC into the skin which, in turn, will act as a depot, releasing the drug in a sustained manner for a prolonged period, while reducing its systemic exposure.

It should be noted that upon removing the MN array patches, no erythema or irritation could be observed, indicating that the drug delivery system is safe. Also, we noticed that the MN shafts were completely disappeared even within 2 h from MN insertion into the skin (the first sampling point), suggesting that the required wearing time for this drug delivery system is minimal.

Also, it is worth mentioning that, after MN application for 2 h, they were completely dissolved and left a thin layer of gel-like residues on the skin surface which consisted of the baseplate polymers and the undelivered MTX NC. These were easily wiped off with a wet cloth.

Taking together the results from both pharmacokinetic and dermatokinetic studies, we can cautiously estimate the amount of MTX delivered by the MN and how this affects the drug`s systemic exposure in comparison with orally administered MTX Na. The mean amount of MTX retained in the skin after 2 h from applying the MN was 624.51 µg/g. This is probably the amount of drug which was delivered by the MN (if we take into consideration the *ex vivo* release studies results, which showed that within the first 3 h from MN application no drug was detected in the receptor solution). This suggests that around 25.1% of the applied dose (2.48 mg/MN array) was delivered. Thus, the amount of drug delivered from the two MN arrays that were applied to the back of each rat would be in the magnitude of 1249.02 µg. This is around 2-fold higher than the orally administered dose (approximately 625 µg/rat). However, if we take into account that MTX bioavailability after oral administration of such a dose to Sprague Dawley rats is reported to be 0.08% (Kuroda et al., 2001), the intradermally delivered dose of MTX (deposited in the skin) by MN would be 24.9-fold higher than the dose delivered by oral administration. Despite delivering such a massive dose into the skin, C_max_ in the MN group was around 40% of that reported in the oral group. This suggests that delivering MTX by MTX NC-MN resulted in a dramatic reduction the systemic drug exposure (*p* < 0.001), indicating that our drug delivery system could help in minimising/avoiding the side effects associated with the systemic administration, thus improving the drug safety.

Du et al. (2019) have demonstrated that delivering around 13.8 µg of MTX Na (once per day) by dissolving hyaluronic-acid-based MN into a psoriasis-like skin inflammation in mice was effective in alleviating the psoriatic lesion and was significantly more efficacious than giving the same drug dose orally. For our MTX NC-MN, the amount of drug delivered from one 0.5 cm^2^ MN array was approximately 624.51 µg. This was around 46-fold higher than the required therapeutically effective dose. Even after 72 h from MN application, the amount of drug retained in the skin was around 78.21 µg. This is still 5.8-fold higher than the reported effective therapeutic dose (Du et al., 2019). This suggests that our drug delivery system could help in improving in the drug efficacy as maintains constant drug concentration at the application for a prolonged time; thus it avoids drug concentration fluctuation which is likely to be produced when the drug is administered once weekly by the conventional administration routes. However, further studies are now required to determine the efficacy of our drug delivery system *in vivo* in a psoriatic skin model.

Although our novel drug delivery system has demonstrated superior properties and capabilities in terms of mechanical strength, insertion properties, dissolution and sustaining the drug release over 72 h, it has some limitations. The first limitation lies in its delivery efficiency, which was relatively low (25.1% of the loaded dose in the MN array). This could be overcome by loading only the delivered amount MTX NC (*i.e.* 624 µg) into the MN tips section that is inserted beneath the *stratum corneum*. This represents around 60% of the MN height as we showed in the MN insertion studies. The second limitation was that our drug delivery system was applied onto a healthy skin model, which is different from psoriatic skin and is often characterised by erythema, scales, red papules and is thickened (Zhao et al., 2020). Therefore, this might possibly affect MN application and drug release kinetics. Although further studies are needed to investigate the efficiency and safety of our drug delivery system in a psoriatic skin model, we are not overly concerned about this issue, as several studies have shown that dissolving MN can successfully penetrate psoriatic skin deliver their payload in mice (Du et al., 2019; Kuroda et al., 2000) and humans (Korkmaz et al., 2015). Additionally, our drug delivery system showed considerable mechanical strength and efficient insertion in both Parafilm M® and excised neonatal porcine skin (Fig. 3(C-F)). We would expect the influence of psoriatic skin on drug release to be minimal, as NC dissolution is typically dependent upon particle size and drug physicochemical properties.

In considering the possibility for self-application of our MTX NC-MN, our research team and others have already published user feasibility studies, with results indicating that MN can be reproducibly applied to the skin without the need for complex applicators or specialist personnel

(Lee et al., 2018; Donnelly et al., 2014). In addition to this, recent work from our laboratory has deduced that larger, multi-array patches (8 cm^2^ MN) can be successfully self-administered by individuals, and to comparable depths as smaller patches with a single array, following appropriate instruction (Ripolin et al., 2017). Moving toward patient usage, comprehensive skin irritation and dermal toxicity evaluations must be carried out, followed by user feasibility studies which will enhance MN concept and design and inform subsequent design approaches for these innovative drug delivery systems.

## 4 Conclusion

Taken together, the results presented here indicate that our unique approach of combining MTX nanocrystals into dissolving MN, leads to a successful intradermal delivery of MTX NC, which in turn acted as a depot releasing the drug in a sustained manner over 72 h. The overriding benefit of the facilitated delivery approach we have reported here, compared to conventional oral administration, lies in the capacity for drug retention in the skin which could potentially lead to improving therapeutic efficacy and minimising MTX systemic exposure, thus avoiding the side effects associated with the conventional systemic administration routes (oral and parenteral administration). Following on from these findings, further studies are now necessitated to investigate the efficacy of our drug delivery system in an *in vivo* psoriatic-skin model.

## CRediT authorship contribution statement

**Ismaiel A. Tekko:** Conceptualization, Methodology, Software, Validation, Formal analysis, Investigation, Data curation, Writing - original draft, Writing - review & editing, Visualization, Project administration. **Andi Dian Permana:** Methodology, Validation, Formal analysis, Investigation, Data curation, Writing - original draft. **Lalitkumar Vora:** Writing - review & editing. **Taher Hatahet:** Software, Writing - review & editing, Visualization. **Helen O. McCarthy:** . **Ryan F. Donnelly:** Investigation, Resources, Writing - review & editing, Supervision, Project administration, Funding acquisition.

## Declaration of Competing Interest

The authors declare no conflict of interest.
